# Enhanced Virus-Specific CD8^+^ T Cell Responses by *Listeria monocytogenes*-Infected Dendritic Cells in the Context of Tim-3 Blockade

**DOI:** 10.1371/journal.pone.0087821

**Published:** 2014-01-31

**Authors:** Cheng J. Ma, Jun P. Ren, Guang Y. Li, Xiao Y. Wu, Dirk G. Brockstedt, Peter Lauer, Jonathan P. Moorman, Zhi Q. Yao

**Affiliations:** 1 Hepatitis (HCV/HIV) Program, Department of Veterans Affairs, James H. Quillen VA Medical Center, Johnson City, Tennessee, United States of America; 2 Department of Internal Medicine, Division of Infectious Diseases, Quillen College of Medicine, East Tennessee State University, Johnson City, Tennessee, United States of America; 3 Aduro BioTech, Inc. Berkeley, California, United States of America; University of Montreal, Canada

## Abstract

In this study, we engineered *Listeria monocytogens* (Lm) by deleting the LmΔ*actA*/Δ*inlB* virulence determinants and inserting HCV-NS5B consensus antigens to develop a therapeutic vaccine against hepatitis C virus (HCV) infection. We tested this recombinant Lm-HCV vaccine in triggering of innate and adaptive immune responses *in vitro* using immune cells from HCV-infected and uninfected individuals. This live-attenuated Lm-HCV vaccine could naturally infect human dendritic cells (DC), thereby driving DC maturation and antigen presentation, producing Th1 cytokines, and triggering CTL responses in uninfected individuals. However, vaccine responses were diminished when using DC and T cells derived from chronically HCV-infected individuals, who express higher levels of inhibitory molecule Tim-3 on immune cells. Notably, blocking Tim-3 signaling significantly improved the innate and adaptive immune responses in chronically HCV-infected patients, indicating that novel strategies to enhance the potential of antigen presentation and cellular responses are essential for developing an effective therapeutic vaccine against HCV infection.

## Introduction

Hepatitis C virus (HCV) infection is a global epidemic characterized by a high rate of chronic infection, poor treatment response, and no available vaccine [1]–[2]. Despite extensive studies on virus-host interactions, it remains unclear why HCV persists in the majority of infected individuals, and more than half of genotype 1 HCV-infected patients fail to respond to standard treatment with pegylated interferon (PegIFN) and ribavirin [1]–[2]. Dendritic cells (DC) are the most potent antigen presenting cells (APC) and serve as an effective means to trigger CTL responses, which are critical to clearing virus [3]–[5]. Impairment of DC and CTL responses by HCV seems to play a pivotal role in the majority (about 85%) of HCV-infected patients who progress to chronic infection; whereas approximately 15% of individuals who spontaneously resolve the acute infection exhibit vigorous innate and adaptive immune responses [4]–[8] - an indication that a vaccine may be attainable.

Since molecular cloning of HCV in 1989, a variety of vaccines have been generated for preclinical trials, including subunit protein or peptide vaccines [9]–[12], recombinant DNA vaccines [13]–[16], recombinant HCV-like particle vaccines [17]–[22], microbial vector vaccines [21]–[24], and more recently, DC-targeting vaccines [25]–[26]. Despite decades of intense research, there is no HCV vaccine available to date and several major obstacles have been identified [27]. Firstly, HCV is highly variable, divided into at least 6 major genotypes and over 100 subtypes. In an infected individual, HCV exists as groups of related but distinct viral populations termed quasispecies [28] that differ in sequence within hyper-variable regions along the genome, making vaccine development extremely difficult. Secondly, there is a lack of appropriate HCV animal models. Apart from humans, the only natural HCV animal model is chimpanzee, a protected species that is costly and hence limited in its availability [29]. HCV genetic mice and cell culture systems have more recently provided important tools for the study of HCV pathogenesis and antiviral agents, including therapeutic vaccines [30]–[31]. Thirdly, there is a shortage of potent immunotherapy (vaccine) vectors. Notably, a distinct *Listeria monocytogenes* (Lm) vaccine platform has been recently developed that specifically deletes the *actA* and *inlB* virulence determinants (*LmΔactA/ΔinlB*). This genetically-modified, live-attenuated *LmΔactA/ΔinlB* vaccine platform effectively segregates toxicity from immunopotency, and is currently being tested in Phase-2 clinical trials in advanced cancers [32]–[38]. Lastly, chronically HCV-infected individuals mount an impaired immune response, partially due to HCV-mediated over-expression of inhibitory receptors, such as programmed death-1 (PD-1) and T cell immunoglobulin mucin domain-3 (Tim-3) on immune cells, which can facilitate viral persistence and vaccine non-responsiveness [39]–[44]. Therefore, it is fundamentally important and clinically applicable to study whether targeting cell negative signaling may improve the efficacy of an Lm-based DC-targeting therapeutic vaccine against HCV infection.

In this study, we explored novel strategies to improve the ability of a live-attenuated Lm-HCV vaccine, generated by inserting the HCV-NS5B consensus antigens into the *LmΔactA/ΔinlB* microbial vector, to trigger DC maturation, antigen presentation, cytokine production, and virus-specific CD8^+^ T cell responses *in vitro* using immune cells derived from HCV-infected and uninfected individuals concomitant with blockade of Tim-3 signaling. Our preliminary data reveal that approaches to improve the potential of antigen presentation and cellular responses are necessary for developing an effective therapeutic vaccine against chronic HCV infection.

## Materials and Methods

### Subjects and Cell isolation

The study protocol was approved by an institutional review board at East Tennessee State University and James H. Quillen VA Medical Center (ETSU/VA IRB, Johnson City, TN), which has contributed to a database for the storage of blood samples from chronically HCV-infected, spontaneously HCV-resolved, or sustained virological response (SVR) 6∼12 months after antiviral therapy, and healthy subjects, for the purpose of viral immunology studies. All participants provided IRB-approved written informed consent to participate in this study, and the demographics of the study group including age, gender, HCV genotype, and viral load were listed in Table 1. Human peripheral blood mononuclear cells (PBMCs) were isolated from the peripheral blood of these subjects by Ficoll-density centrifugation with lympho-H (Atlanta biological, Lawrenceville, GA). CD14^+^ M/M_Ø_ and CD3^+^ T cells were purified from PBMCs by magnetic beads with column purification according to the manufacturer's instructions (purity >95%; Miltenyi Biotec Inc, Auburn CA). CD14^+^ M/M_Ø_ were used for DC induction, and CD3^+^ T cells were frozen in liquid nitrogen in RPMI freezing medium containing 50% FBS and 10% DMSO for CTL assays. The cells were cultured with RPMI 1640 containing 10% fetal bovine serum (FBS, Life Technologies, Gaithersburg, MD), 100 µg/ml penicillin-streptomycin (Thermo Scientific Logan, Utah), and 2 mM L-glutamine (Thermo Scientific, Logan, Utah) at 37°C with 5% CO_2_ atmosphere.

**Table 1:** Demographics of the study group.

Group	Number	Age (mean)	Gender	Genotype	Viral load (IU/ml)
**HCV-infected**	40	28∼66 (53)	39M/1F	1 (a/b)	3,980∼50,000,000
**HCV-SVR**	10	33∼64 (51)	10 M	1 (a/b)	undetectable
**HCV-SR**	6	41∼63 (54)	6M	Unknown	undetectable
**HS**	12	25∼58 (42)	8M/4F	N/A	N/A

SVR: sustained virological response 6∼12 months following antiviral therapy; SR: HCV spontaneously resolved; HS: healthy subjects; M/F: male/female; N/A: not applicable.

### Bacterial Strains

**Figure 1:**
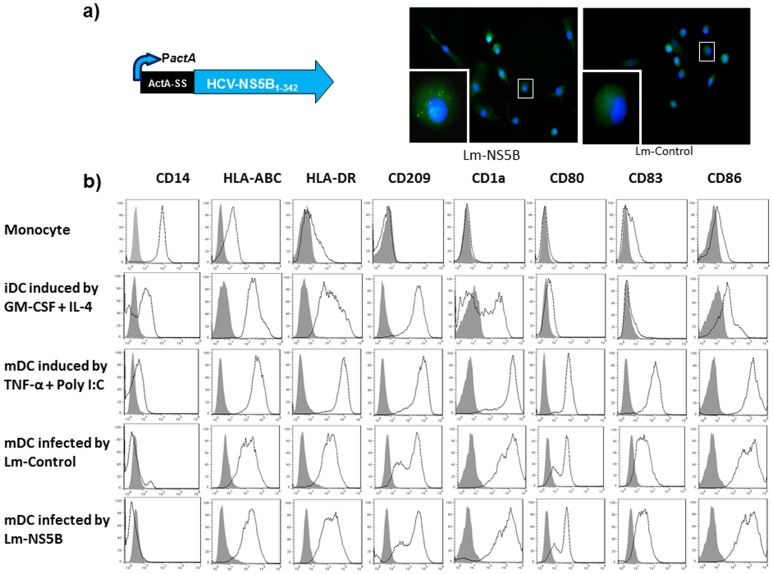
Lm-based HCV vaccine can naturally infect monocyte-derived DC and drive their maturation. a) Lm HCV-NS5B antigen expression cassette and HCV antigen expression in Lm-NS5B-infected DC. Lm-HCV vaccine was generated by inserting consensus HCV-NS5B antigens in the *LmΔactA/ΔinlB* vector. M/M_Φ_ isolated from healthy subjects (HS) were cultured in the presence of GM-CSF and IL-4 for 5 days to induce immature DC (iDC), and then infected by live attenuated Lm-NS5B or Lm-control for 2 days to induce mature DC (mDC). The expression of HCV-NS5B antigen was detected by immunofluorescence staining, in conjunction with DAPI nuclear staining, and observed by fluorescence microscopy as described in the Methods. The imaging was taken at 20× magnification and typical cell (squares) with or without HCV antigen expression in DC was further amplified in the left lower corner. b) Expression of cell differential surface markers. Purified monocytes were cultured in the presence of GM-CSF and IL-4 for 5 days to induce iDC, and then infected by live attenuated Lm-NS5B or Lm-control for 2 days to induce mDC. TNF-α and polyI:C served as positive DC maturation inducers. The expressions of CD14, HLA-ABC, HLA-DR, CD209, CD1a, CD80, CD83, CD86, on the surface of monocyte, GM-CSF/IL-4-induced iDC, TNF-α/PolyI:C-induced or Lm-control and Lm-NS5B-induced mDC were analyzed by flow cytometric analysis. Data are shown as overlaid histograms of the cell surface marker expression with isotype control in grey-filled histogram. The results are reproducible using M/M_Φ_ isolated from multiple HS or HCV-infected individuals.

Live attenuated Lm strain expressing HCV-NS5B antigens was constructed by cloning the consensus sequence encoding amino acids 1–342 of HCV-NS5B under control of the actA promoter and in-frame with the amino terminus of the actA protein into a derivative of the pPL2 integration vector and integrating the construct at the *tRNA* ^Arg^ locus of the *LmΔactAΔinlB* chromosome as described [32] (Fig. 1a). Empty Lm vector served as control. Experimental stocks were prepared by growing bacteria to early stationary phase, washing in phosphate buffered saline (PBS), resuspending to approximately 1×10^10^ colony-forming units per ml, and freezing aliquots at −80°C for later use.

### DC induction and infection

Monocyte-derived immature DC (iDC) were induced by incubation of 1×10^5^/500 µl/well CD14^+^ monocytes in a 24 well plate with GM-CSF and IL-4 (40 ng/ml and 20 ng/ml respectively, PeproTech, Rocky Hill, NJ) for 5 days. iDC were infected by Lm-NS5B or Lm-control with an MOI of 10 in a 5 ml Eppendorf tube shaking at 225 rpm 37°C for 1 h; the infected cells were washed with RPMI at 1000 rpm for 5 min twice, and then cultured in a 24-well plate in the complete RPMI containing gentamycin (50 µg/ml, Sigma, St Louis, MO) for 2 days to mature. TNF-α (10 ng/ml, Miltenyi Biotec Inc, Auburn CA) and poly I:C (50 µg/ml, Amersham Biosciences, Piscataway, NJ) were used as positive inducers for DC maturation.

### DC antigen uptake

Antigen uptake by DC was performed as previously described [45]–[46]. Briefly, FITC-labeled bovine serum albumin (50 µg/ml BSA-FITC; Sigma, St Louis, MO) was added to 1×10^6^ cells at 37°C. Uptake of the same concentration of BSA-FITC at 4°C served as a negative control. Both groups were incubated for 30 minutes in the dark. After incubation, cells were washed twice in cold PBS plus 1% BSA and analyzed by flow cytometry. Data represent the difference in percentage of positive cell and corrected mean fluorescence intensity (ΔMFI) of DC BSA-FITC uptake at 37°C and 4°C.

### Lm-HCV-DC-induced T cell response

Lm-infected DC were co-cultured with autologous CD3^+^ T cells in the presence of IL-2 (10 ng/ml, eBioscience, San Diego, CA) for 5 days, followed by flow cytometric analysis for surface staining of FITC-CD3, PerCP-CD8, and HCV-NS5B tetramer (ALYDVVSKL, synthesized by PEPTIDE 2.0, Chantilly, VA; Tetramer prepared by NIH Tetramer Core Facility, Atlanta, GA). Cells isolated from HCV spontaneously resolved or IFN/RBV-treated individuals with SVR and HS without peptide priming were used as positive and negative subject controls. Empty Lm vector-infected DC and HCV-NS5B consensus CTL peptide-load DC served as negative and positive reagent controls.

### Flow cytometry & Immune fluorescence

For detecting cell surface markers, purified human CD14^+^ M/M_Φ_, TNF-α/IL-4-induced iDC, GM-CSF/Poly I:C-induced or Lm-induced mDC, CD3^+^ T cells were subjected to flow cytometric analysis using FITC-CD14, PE-HLA-ABC, PerCP-HLA-DR, PE-CD209, APC-CD1a, FITC-CD80, APC-CD83, PE-CD86 from Miltenyi Biotec Inc (Auburn CA), and APC-Tim-3 from eBioscience (San Diego, CA, clone F38-2E2). To measure intracellular IL-12 expression, purified M/M_Ø_, TNF-α/IL-4-induced iDC, and Lm-infected mDC were stimulated by 1 µg/ml of LPS and 2.5 µg/ml of R848 (Santa Cruz) in the presence of Brefeldin A (BioLegend, San Diego, CA) forbidding cytokine secretion for 6 h, followed by intracellular staining with APC-IL-12p70 (eBioscience). To measure intracellular IFN-γ and granzyme-B expression by HCV-specific CTLs, CD3^+^ T cells were incubated with Lm-infected DCs in the presence of IL-2 for 5 days and stimulated by 100 ng/ml of PMA and 1 µg/ml of Ionomycin (InvivoGen, San Diego, CA) with Brefeldin A added 6 h prior to harvest the cells, followed by cell surface staining with PE-NS5B-tetramer, FITC-CD3, PerCP-CD8, and intracellular staining with APC-IFN-γ or APC-granzyme-B (eBioscience). The intracellular cytokine staining was carried out using Inside Stain kit (Miltenyi Biotec) per manufacturer's instructions. For gating strategy, isotype-matched control antibodies (eBioscience) and fluorescence minus one (FMO) controls were used to determine the background levels of staining and adjust multicolor compensation. The cells were analyzed on a FACS Calibur flow cytometry (BD, Franklin Lakes, NJ) and FlowJo 7.6.1. (Ashland, OR).

HCV antigen expression in Lm-NS5B-infected DC was carried out by immune staining with 1∶10 HCV-NS5B mouse monoclonal antibody (LifeSpan Biosciences, Inc. Seattle, WA) overnight after fixation and permeabilization of cells, followed by staining with 1∶2000 Alexa Fluor@ 488-conjucated donkey anti-mouse secondary antibody (Life Technology, Grand Island, NY) for 1 h, and then observed by AMG fluorescence phase microscope (Bothell, WA).

### Tim-3 blockade

Purified CD14^+^ M/M_Ø_ or CD3^+^ T cells were incubated with LEAF™ anti-human Tim-3 antibody (10 µg/ml, BioLegend, clone F38-2E2) or control IgG, followed by stimulation and subjected for flow cytometric analysis, as described in the result.

### Statistical analysis

Study results are summarized for each group and results are expressed as the mean ± standard deviation (SD). Comparison between two groups is performed by SPSS-18 software. Comparisons of data between groups were made using one-way analysis of variance (ANOVA), and Tukey's procedure for multiple-range tests was performed. Wilcoxon pair t-test is used to compare the significance of changes in Tim-3 blocking experiments. Values of p<0.05 (*), p<0.01(**), p<0.001 (***) were considered significant or very significant. NS = no significance.

## Results

### Lm-based HCV vaccine can infect monocyte-derived DC and drive their maturation

DCs are instrumental for the initiation of immune responses to foreign antigens because of their professional competence to capture and present antigens to T cells; however, they must be activated to maturity before serving as efficient APCs [3]. As an initial approach exploring novel strategies to augment the efficacy of an Lm-based DC-targeting vaccine against HCV infection, we first examined whether this Lm-based HCV vaccine can infect DC, express HCV antigens, and drive DC maturation. To this end, monocytes were isolated from HCV-infected and healthy subjects (HS), cultured in the presence of granulocyte-macrophage colony-stimulating factor (GM-CSF) and IL-4 for 5 days to induce immature DC (iDC), and then infected by live-attenuated Lm-NS5B or Lm-control for 2 additional days to induce mature DC (mDC). Tumor necrosis factor-α (TNF-α) and Poly I:C served as positive DC maturation inducers. The expressions of HCV antigens and maturation markers by DC were analyzed by immunofluorescence and immunophenotyping. This Lm-based vaccine is unique in that it can naturally target human DC [36] and express HCV antigens in the cytoplasmic compartments of the infected cells (Fig. 1a, infectivity >90%). Following initial induction and Lm infection, resting monocytes are activated to lose surface CD14 marker, but concurrently express HLA and co-stimulatory molecules such as HLA-ABC, HLA-DR, CD209, CD1a on iDC, and subsequently maturation markers such as CD80, CD83, and CD86 on mDC (Fig. 1b). Notably, the process of DC maturation mediated by Lm-HCV or Lm-control is comparable in terms of efficiency and phenotypic development when compared to induction by TNF-α and Poly I:C, the standard DC maturation stimuli. No significant differences are observed in the susceptibility of DC to Lm-NS5B or Lm-control infection as well as the expression levels of these cell surface markers in HCV-infected individuals versus HS (data not shown). These results suggest that this Lm-HCV strain can naturally target human DC, activate DC maturation, present HCV antigens, and serve as a potential DC-targeting HCV vaccine.

### Lm-HCV-DC-induced virus-specific CTL activation is diminished and negatively correlates with Tim-3 expression

**Figure 2:**
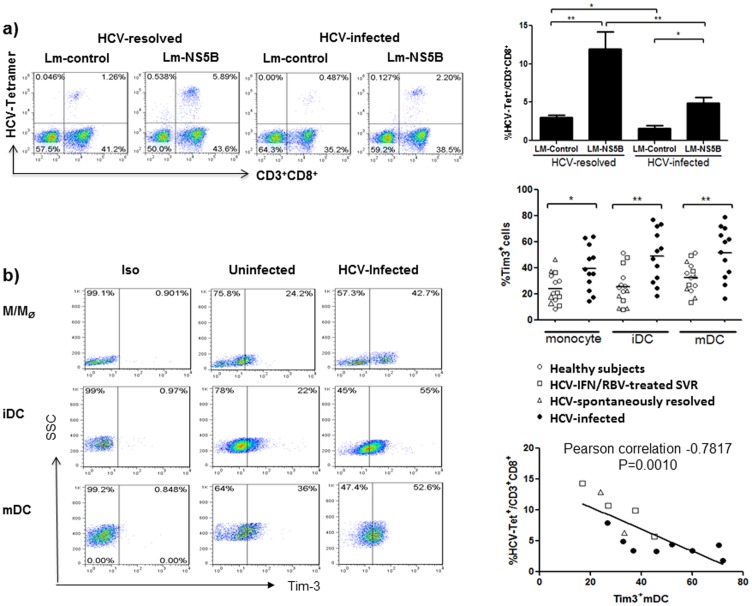
Diminished virus-specific immunity induced by Lm-HCV-infected DCs negatively correlates with Tim-3 expression. a) Lm-based DC-targeting HCV vaccine only elicits a weak CTL response using immune cells from chronically HCV-infected individuals. M/M_Φ_ isolated from HCV-infected as well as HCV-resolved individuals were induced by GM-CSF and IL-4 for 5 days to iDC, which were subsequently infected by Lm-NS5B or Lm-control for 2 additional days to mDC; and then incubated with autologous T cells in the presence of IL-2 for another 5 days to generate CTL. Activation of the virus-specific CTLs was assessed by HCV-NS5B tetramer staining of CD3^+^CD8^+^ T cell frequencies as described in the Methods. Data are shown as representative dot plots of the staining with percentages of cells in each quadrant (left), and the mean ± SD of the percentages of HCV-tetramer^+^ CTLs (HCV-Tet^+^) in CD3^+^CD8^+^ T cells challenged by Lm-control or Lm-NS5B-infected DCs from 6 HCV-resolved (2 spontaneously resolved and 4 HCV-treated SVR subjects) and 8 HCV-infected individuals (upper right); *P<0.05, **P<0.01. b) Tim-3 expression on M/M_Φ_, iDC, and mDC induced from HCV-infected and uninfected individuals. M/M_Φ_ were isolated from HCV-infected and uninfected individuals, iDC were induced by stimulation with GM-CSF/IL-4 for 5 days, and mDC were induced by Lm-infection for 2 days. Tim-3 expressions on M/M_Φ_, iDC, and mDC were carried out as described in the Methods. Isotype-matched control antibodies and fluorescence minus one (FMO) controls were used to determine background levels of staining and adjust multicolor compensation as gating strategy. Data are shown as representative dot plots with percentages of cells in the gated area (left), and summary with mean of the percentages of Tim-3^+^ cells in M/M_Φ_, iDC, and mDC from 14 HCV-resolved (4 spontaneously resolved, 4 HCV-treated SVR subjects, 6 HS) and 12 HCV-infected individuals (middle right). The relationship between Tim-3 expression on mDC and percentage of HCV-specific tetramer staining on CD3^+^CD8^+^ CTLs was analyzed by Pearson correlation (lower right). Each symbol represents one subject, and different individuals were shown by different symbols (^O^ for HS, ^Δ^ for SR, ^□^ for SVR, and ^•^ for HCV-infected subject); *P<0.05, **P<0.01.

Presumably, Lm-HCV-infected DC will express HCV antigens in the appropriate cytoplasmic compartments, resulting in HCV proteins having access to MHC class I molecules for presentation to CD8^+^ T cells. However, the antigen-presenting capacity of DC and the cytotoxic activity of CD8^+^ T cells are known to be diminished in the setting of HCV infection, leading to immune tolerance and viral persistencc [4]–[8]. To assess if Lm-HCV-infected DC are able to stimulate virus-specific memory T cell responses and thus potentially function as a therapeutic approach to break through immune tolerance during chronic HCV infection, monocytes isolated from HCV-infected as well as HCV-resolved individuals were induced to iDC by GM-CSF and IL-4 treatment for 5 days, and subsequently infected by Lm-NS5B or Lm-control for 2 additional days to induce mDC; then incubated with autologous T cells in the presence of IL-2 for another 5 days to generate CTL. Activation of virus-specific CTLs was assessed by HCV-NS5B tetramer staining of CD3^+^CD8^+^ T cell frequencies. As shown in Fig. 2a, a baseline of HCV-specific tetramer staining is detected in T cells incubated with Lm-control-infected DC, indicating an antigen-independent activation of CD3^+^CD8^+^ T cells by Lm-infected DC. However, a higher frequency of HCV-tetramer^+^ is observed in CD3^+^CD8^+^ T cells stimulated with DC harboring Lm-NS5B than those with Lm-control vector, supporting the notion that additional HCV antigen triggering is necessary for an effective memory T cell response to an Lm-DC-targeting vaccine. Notably, strong CTL activation are elicited with Lm-NS5B-infected DC and T cells derived from HCV-resolved individuals, including those with spontaneous resolution of HCV infection and those with sustained virological response (SVR) after PegIFN/ribavirin treatment. In contrast, HCV-tetramer^+^ frequencies in CD3^+^CD8^+^ T cells triggered by Lm-NS5B-DCs are significantly lower in chronically HCV-infected patients than those HCV-resolved individuals. Of note, this Lm-NS5B-DC-targeting vaccine can only induce virus-specific T cell activation in limited, but not all, HCV-infected patients (data not shown), suggesting either limited antigenic coverage of Lm-NS5B or possible viral mutational escape of immune surveillance - an indications for multi-epitopic or polyvalent vaccines to break through the immune tolerance in the setting of chronically HCV infection.

Tim-3 has been shown to be crucial in dampening innate and adaptive immune responses during HCV infection [40]–[44]. To investigate the underlying mechanisms of the diminished CTL response induced by Lm-based DC-targeting HCV vaccine in the setting of chronic HCV infection, we examined the expression of Tim-3 on monocyte-derived DC from HCV-infected versus uninfected subjects. As shown in Fig. 2b, Tim-3 expression is significantly higher in M/M_Ф_, as well as GM-CSF/IL-4-induced iDC, and Lm-induced mDC from chronically HCV-infected patients than in uninfected individuals, including those naturally HCV-resolved subjects, HCV-treated SVR subjects, and HS. This is true when separating the HS in the uninfected group as control for statistical analysis. Additionally, Tim-3 expression is also observed at a higher frequency on the Lm-DC-activated CTLs from HCV-infected than HCV-resolved subjects (Fig. 5a). Importantly, the levels of Tim-3 expression on mDC negatively correlated with the percentages of HCV-specific tetramer staining on CD3^+^CD8^+^ CTLs by Pearson correlation analysis (Fig. 2b). These results suggest that Tim-3 expression on innate DC is directly linked to the blunted adaptive CTL activation triggered by Lm-HCV vaccine.

### Antigen uptake, maturation, and IL-12 production by DCs are improved with Tim-3 blockade

**Figure 3:**
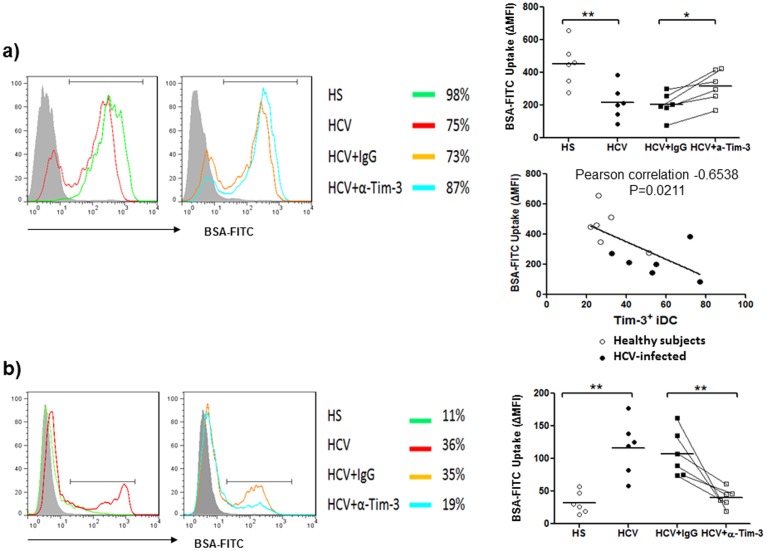
Tim-3-depedent antigen uptake by monocyte-derived DCs in HCV-infected and uninfected subjects. a) Tim-3-dependent antigen uptake by monocyte-derived immature DC (iDC) in HCV-infected and uninfected individuals. M/M_Ø_ isolated from HCV-infected and HS were induced to iDC by stimulation with GM-CSF and IL-4 in the presence of anti-Tim-3 or control IgG antibodies for 5 days. The capacity of antigen phagocytosis by iDC was assessed by measuring iDC uptake of BSA-FITC by flow cytometry as described in the Methods. Data are shown as representative histograms of antigen-uptake DC in different subjects and treatments overlaid with isotype control in the grey-filled histogram (left), and the summary of corrected mean fluorescence intensity (ΔMFI) of iDC BSA-FITC uptake at 37°C and 4°C from multiple subjects (upper right); *P<0.05, **P<0.01. The relationship between antigen uptake and Tim-3 expression on iDC in various subjects was analyzed by Pearson correlation (middle right). Each symbol represents one subject, the mean value is shown and the same individual treated with control IgG or anti-Tim3 is line-connected. b) Tim-3-dependent antigen uptake by monocyte-derived mature DC (mDC) in HCV-infected and uninfected individuals. M/M_Ø_ isolated from 6 HCV-infected and 6 HS were induced to iDC by stimulation with GM-CSF and IL-4 in the presence of anti-Tim-3 or control IgG antibodies for 5 days, and further induced to mDC by Lm infection for additional 2 days in the presence of blocking or control antibodies. The capacity of antigen phagocytosis by mDC was carried out in the same way. Data are shown as the overlaid histograms with isotype control in the grey-filled histogram (left), the percentages of antigen-uptake cells in different subjects and treatments (middle), and the summaries of ΔMFI of BSA-FITC uptake by mDC from multiple subjects (right). Each symbol represents one subject; **P<0.01.

iDC capture antigen efficiently but lose this capacity upon maturation, and thus a decrease of antigen uptake has been used as a functional assay to monitor DC maturation [45]–[46]. Here we tested the uptake of a labeled antigen (BSA-FITC) by the GM-CSF/IL-4-induced iDC and Lm-NS5B-induced mDC from HCV-infected and uninfected subjects in the presence or absence of Tim-3 blocking antibody or control IgG. As shown in Fig. 3a, iDC induced from patients with chronic HCV infection capture less antigen than those from HS, an effect that can be improved by blocking Tim-3 signaling. Importantly, antigen uptake by iDC was found to be negatively correlated with the level of Tim-3 expression on these cells. After Lm infection and DC maturation, however, mDC from HS lost their ability to uptake antigens. In contrast, mDC from patients with chronic HCV infection continued to capture BSA-FITC – an indication of an immature status – and this can be corrected by Tim-3 blockade (Fig. 3b). Cumulative results of antigen uptake, shown as corrected mean fluorescence intensity (ΔMFI) of BSA-FITC uptake by iDC and mDC induced from monocytes derived from multiple HCV-infected versus uninfected individuals, demonstrate a significant difference in the capacity of DC phagocytosis in a Tim-3-dependent manner.

**Figure 4:**
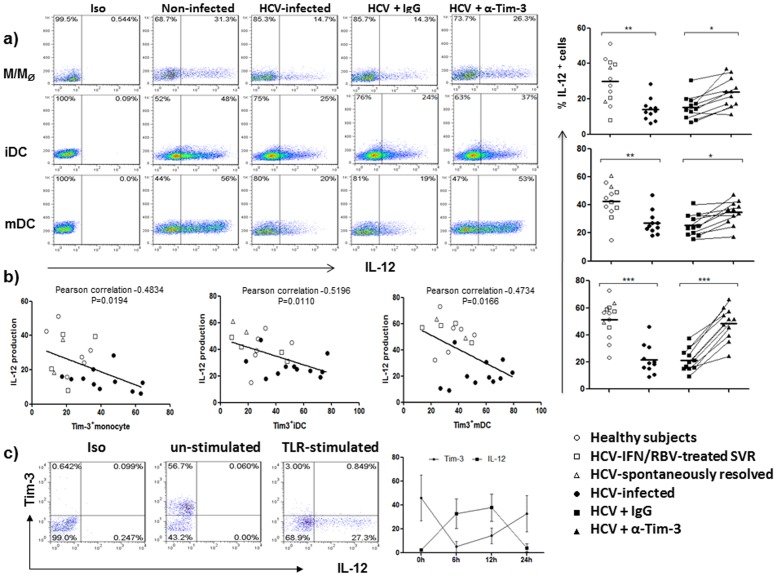
Tim-3 blockade improves TLR-mediated IL-12 expression by monocyte-derived DC from HCV-infected individuals. a) IL-12 expression by monocyte-derived DCs from HCV-infected patients is impaired and can be improved by blocking Tim-3 signaling. M/M_Ø_ isolated from HCV-infected and uninfected individuals were induced to iDC by GM-CSF/IL-4 stimulation for 5 days, and further induced to mDC by Lm infection for 2 days, in the presence of anti-Tim-3 or control IgG antibodies. Intracellular IL-12 expression was analyzed, following LPS/R848 stimulation for 6 h, by flow cytometry as described in the Methods. Isotype-matched control antibodies and fluorescence minus one (FMO) controls were used to determine background levels of staining and adjust multicolor compensation as gating strategy. Data are shown as representative dot plots with percentages of positive cells in the gated area (left), and summary of the percentage of IL-12 expression levels with mean value on M/M_Ø_, iDC, and mDC induced from 11 HCV-infected and 12 uninfected individuals, including 2 spontaneously HCV-resolved, 4 HCV-treated SVR subjects, and 6 HS (right). Each symbol represents one subject, the same subject treated with control IgG or anti-Tim3 antibodies is line-connected, and the legend of subjects with different treatment is shown below; *P<0.05, **P<0.01, ***P<0.001. b) Relationship between IL-12 and Tim-3 expressions by M/M_Ø_, iDC, and mDC in various subjects is analyzed by Pearson correlation. Each symbol represents one particular subject, different individuals were separated by different symbols (^O^ for HS, ^Δ^ for SR, ^□^ for SVR, and ^•^ for HCV-infected subject); and statistical analysis is shown on top of each figure. c) Relationship between Tim-3 and IL-12 expressions by M/M_Ø_ following LPS/R848 stimulation. M/M_Ø_ isolated from HCV-infected individuals were stimulated with LPS/R848 for specified times, cell surface Tim-3 and intracellular IL-12 expressions were analyzed by flow cytometry. Data are shown as representative dot plots of isotype staining, Tim-3 versus IL-12 expressions in un-stimulated, and TLR-stimulated M/M_Ø_ at 6 h, as well as summary mean ± SD from multiple subjects (n = 6) at various time points following stimulation.

As professional APCs, DCs secrete high levels of interleukin-12 (IL-12), a key Th1 polarizing cytokine that promotes CTL responses [47]. Therefore, we next examined the effect of Tim-3 on IL-12p70 expression by M/M_Ф_ and DC induced from HCV-infected versus uninfected individuals following stimulation with TLR4 ligand-LPS and TLR7/8 ligand-R848, which are essential for IL-12 expression [48]–[49]. In contrast to the higher levels of Tim-3 expression, M/M_Ф_ as well as iDC and mDC from chronically HCV-infected patients exhibit lower levels of IL-12 production compared to uninfected individuals, including spontaneously HCV-resolved, HCV-treated SVR subjects, and HS. Again, this is true when separating the HS in the uninfected group as control for statistical analysis. Importantly, blocking Tim-3 signaling can significantly improve IL-12 production by M/M_Ф_ and DC from HCV-infected individuals (Fig. 4a representative dot plots on the left and summary data from multiple subjects on the right).

**Figure 5:**
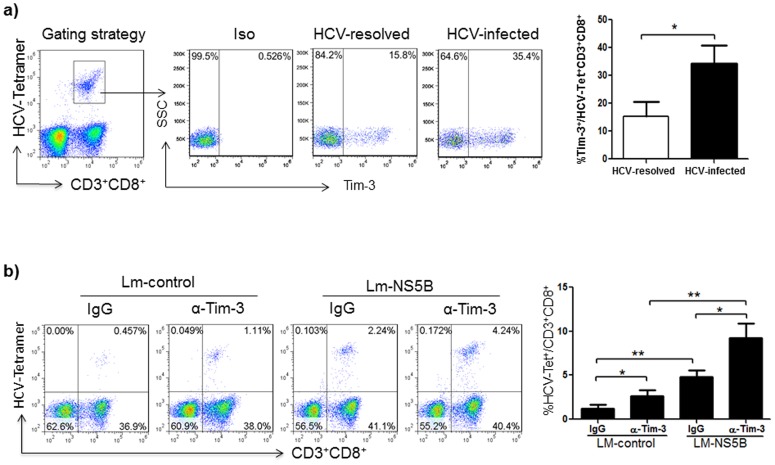
Virus-specific CD8^+^ T cell activation by Lm-HCV-DC challenge with Tim-3 blockade. a) Tim-3 expression on virus-specific CD8^+^ T cells from HCV-infected and resolved individuals. M/M_Ø_ isolated from HCV-infected and HCV-resolved subjects were stimulated with GM-CSF and IL-4 for 5 days to induce iDC, which were infected with Lm-NS5B for 2 days to induce mDC, and then incubated with autologous T cells in the presence of IL-2 for 5 days to activate CTLs. The cells were immune-staining with APC-Tim-3, FITC-CD3, PerCP-CD8, and PE-HCV-NS5B-tetramer (HCV-Tet). Data are shown as representative dot plots of gating strategy for Tim-3 expression on HCV-Tet+CD3+CD8+ CTLs on the left, and mean ± SD from 10 HCV-infected versus 6 HCV-resolved subjects (2 spontaneously resolved and 4 HCV-treated SVR subjects) on the right; *P<0.05. b) HCV-specific CD8^+^ T cell activation by Lm-HCV-DC challenge concomitant with Tim-3 blockade. M/M_Ø_ isolated from HCV-infected patients were stimulated with GM-CSF and IL-4 for 5 days to induce iDC, which were infected with Lm-control or Lm-NS5B for 2 days to induce mDC, and then incubated with autologous T cells in the presence of IL-2 for 5 days to activate CTLs. The cultures were carried out in the presence of anti-Tim-3 or IgG antibodies during induction of iDC and mDC as well as activation of T cells. HCV-specific T cell activation was assessed by measuring the frequencies of HCV-NS5B-tetramer^+^ CTLs (HCV-Tet^+^) in CD3^+^CD8^+^ T cells. Data are shown as representative dot plots of the tetramer^+^ frequencies in the gated CD3^+^CD8^+^ T cells (left), and summary of mean ± SD from 6 HCV-NS5B tetramer^+^ patients; *P<0.05, **P<0.01.

M/M_Ф_, the primary source for DC development, have been shown to be impaired in IL-12 production through Tim-3 signaling during HCV infection [44]. As we have previously reported, there is no correlation between the levels of HCV RNA and Tim-3 expression; however, a negative correlation is identified between Tim-3 and IL-12 expressions by M/M_Ф_, iDC, or mDC in HCV-infected as well as uninfected individuals (Fig. 4b). To further address the role of Tim-3 in controlling IL-12 expression, we also dynamically compared the relationship between Tim-3 expression and IL-12 production by M/M_Ф_ from HCV patients. As shown in Fig. 4c, Tim-3 is highly expressed on the surface of resting M/M_Ф_, whereas IL-12 is barely detectable in the un-stimulated M/M_Ф_, suggesting that Tim-3 may function as a cap or brake for TLR-mediated IL-12 production [50]. Following TLR stimulation, Tim-3 rapidly declines from the cell surface (within 6 h), allowing TLR-driven IL-12 expression, which peaks at 12 h and exhausts at 24 h, by which time Tim-3 is recycled or expressed again on the cell surface. Notably, IL-12 is primarily expressed by Tim-3 negative cells. This kinetic relationship between cell surface Tim-3 and intracellular IL-12 expressions is also observed in GM-CSF-induced iDC and Lm-induced mDC, which exhibit lower Tim-3 expression but higher IL-12 production (data not shown), indicating that Tim-3 negatively controls IL-12 expression in innate APCs.

### Lm-HCV-DC-induced virus-specific CD8^+^ T cell responses are improved by Tim-3 blockade

The key to eradicating acute HCV infection lies in eliciting effective innate and adaptive immunity, especially virus-specific CTL responses [3]–[5]. In contrast, chronic HCV infection is characterized by a low CTL frequency, weak CTL cytokine production, and exhausted CTL killing activity, in conjunction with a high level of Tim-3 expression [40]–[41]. Here we show that Tim-3 is expressed at higher levels on monocyte-derived DC as well as Lm-HCV-DC-activated CTLs from HCV-infected patients than in HCV-resolved individuals (Fig. 2b and 5a). To determine if Lm-HCV-infected DC concomitant with Tim-3 blockade may elicit improved virus-specific T cell immunity and thus provide a novel therapeutic approach for chronic HCV infection, M/M_Ф_ derived from patients with HCV infection were induced by GM-CSF and IL-4 for 5 days to iDC, infected by Lm-NS5B or Lm-control for 2 days to mDC, and then incubated with autologous T cells for additional 5 days to induce memory CTL responses. Tim-3 blocking or control IgG antibodies were added during the induction of iDC and mDC as well as incubation with T cells. The above treated T cells were then subjected to detection of HCV-specific CD8^+^ T cell activation, cytokine production, and killing activity by HCV-NS5B-tetramer staining in the context of intracellular IFN-γ and Granzyme-B expression by CD3^+^CD8^+^ T cells. As shown in Fig. 5b, a baseline HCV-specific tetramer staining is detected in T cells incubated with Lm-control-infected DC in the presence of the IgG control antibody, indicating a general activation of CD3^+^CD8^+^ T cells by Lm-infected DC. However, greater HCV-specific CTLs of HCV-infected patients are activated by Lm-NS5B-DC than those triggered with Lm-control-DC, which is consistent with the results shown in Fig. 2A, suggesting that DC presenting HCV antigens can trigger finite levels of virus-specific CTL response with cells from HCV-infected patients. Notably, blocking Tim-3 signaling significantly improves HCV-specific CTL activation, with T cells in the context of Lm-control-DC and especially of Lm-NS5B-DC (Fig. 5b α-Tim-3 arm, representative dot plots on the left and summary data from multiple subjects on the right).

**Figure 6:**
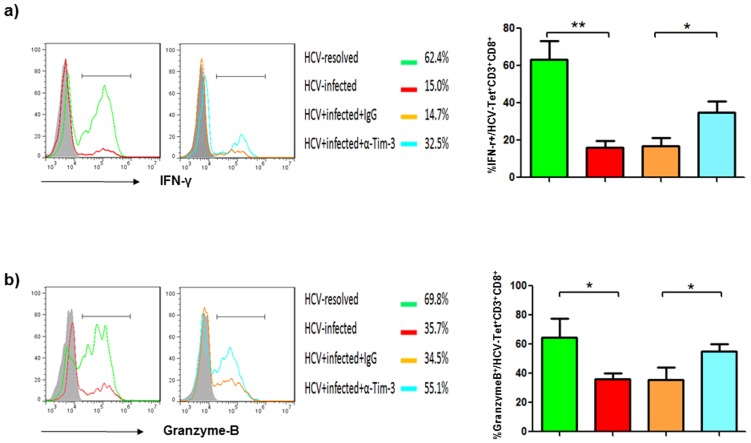
Improved HCV-specific CD8^+^ T cell responses by Lm-HCV-DC challenge and Tim-3 blockade. a) IFN-γ production by HCV-specific CTLs induced from HCV-resolved and HCV-infected individuals in the presence or absence of anti-Tim-3 or IgG antibodies. Lm-HCV-DC-mediated HCV-specific CTL activation was carried out and intracellular IFN-γ production measured as described in the Methods. Data are shown as representative histograms of IFN-γ^+^ cells in HCV-Tet^+^CD3^+^CD8^+^ CTLs overlaid with isotype control in the grey-filled histogram (left), the percentages of IFN-γ-producing cells in the different subjects and treatments (middle), and the mean ± SD of percentages IFN-γ^+^ cells in HCV-tet^+^CD3^+^CD8^+^ CTLs from 6 HCV-infected and 6 HCV-resolved subjects (right); *P<0.05, **P<0.01. b) Granzyme-B production by HCV-specific CTLs induced from HCV-resolved and HCV-infected individuals in the presence or absence of anti-Tim-3 or IgG antibodies. Lm-HCV-DC-mediated HCV-specific CTL activation was carried out as above and intracellular Granzyme-B production measured as described in the Methods. Data are shown as the overlaid histograms with isotype control in the grey-filled histogram (left), the percentages of Granzyme B-producing cells in the different subjects and treatments (middle), and the summaries of percentages Granzyme-B^+^ cells in HCV-tet^+^CD3^+^CD8^+^ CTLs from 6 HCV-infected versus 6 HCV-resolved subjects (right); *P<0.05.

In addition to CTL activation, we also assessed IFN-γ and Granzyme-B productions by HCV-specific CTLs challenged with Lm-HCV-DC in the presence of anti-Tim-3 or IgG antibodies. As shown in Fig. 6a and 6b, strong IFN-γ and Granzyme-B expressions by HCV-specific CD3^+^CD8^+^ T cells are detected with immune cells derived from HCV-resolved individuals, including spontaneously HCV-resolved as well as HCV-treated SVR subjects; whereas diminished CTL responses are observed with cells from chronically HCV-infected patients. Importantly, the impaired CTL responses induced by Lm-HCV-DC can be improved by blocking Tim-3 signaling during the process of DC induction and T cell activation. These results suggest that both antigen presentation and cellular response capacities in the phase of innate and adaptive immunity are essential for generating an effective therapeutic vaccine against HCV infection.

## Discussion

DC and CTL dysfunctions are characteristic of and reasons for the chronic infection, poor treatment response, and lack of effective vaccine during HCV infection. In this report, we provide pilot evidences that demonstrate: 1) Lm-based HCV vaccine is unique in that it can naturally target human DC, thereby driving DC maturation and presenting HCV antigens; 2) Lm-based HCV vaccine can only elicit a weak DC and T cell responses in chronically HCV-infected individuals, who present higher levels of Tim-3 expression on immune cells; 3) blocking Tim-3 signaling can significantly improve innate to adaptive immune responses *ex vivo* using cells from chronically HCV-infected patients, indicating that challenge with an Lm-based DC-targeting HCV vaccine along with blockade of Tim-3 signaling may be a viable approach to boosting immune responses against HCV infection.

A novel approach of this study is to use genetically modified Lm expressing viral antigens in DC as a therapeutic vaccine to challenge memory T cell responses against HCV infection. Specifically, we have tested the efficacy of a new Lm-HCV vaccine that expresses a consensus-sequence of HCV-NS5B antigens *ex vivo* in immune cells derived from HCV-infected versus uninfected or HCV-resolved individuals. Since a major challenge of HCV vaccine development is ineffective viral gene delivery in an appropriate testing model system [27], our study using an Lm vector to deliver HCV antigens targeting DC and T cells from virally infected patients may have more clinical applicability as a new method of immunotherapy. Lm's unique attributes makes it an ideal vector for the development of HCV therapeutic vaccines [32]–[38]: i) it has a good safety profile in both preclinical animal models as well as humans in multiple clinical trials; ii) it naturally infects DC, efficiently presents target antigens to both MHC class I and class II molecules, and effectively stimulates both innate and adaptive immune responses; iii) it activates TLRs and non-TLRs, down-regulates PD-1 and Tim-3, and thus has the potential to overcome immune exhaustion by an antigen-dependent as well as antigen-independent mechanisms in the setting of chronic infections. Thus, live-attenuated LmΔactA/ΔinlB vaccines may be a useful platform for prophylactic vaccination in healthy subjects and for targeted immunotherapy in patients with cancer or infectious diseases [32]–[38]. Here we show that this recombinant Lm-NS5B strain is an effective DC-targeting vaccine with potential to trigger HCV-specific CTL memory responses using immune cells from HCV-infected or HCV-resolved individuals. One limitation of this specific recombinant vaccine is its narrow antigenic coverage, or possibly antigenic escape by an immunodominant strain of HCV in chronically infected individuals, in that it can only trigger virus-specific CTL responses in limited but not all HCV-infected patients. Given that acute HCV clearance has been correlated with a vigorous T cell response encompassing a large number of viral epitopes, an Lm-based DC-targeting vaccine expressing multiple epitopes of HCV prevalent in different populations is clearly worth pursuing.

Active immunotherapy targeting DCs has shown great promise in animal models and in clinical trials for the treatment of human diseases. Provenge® (Sipuleucel-T), which consists of antigen-loaded DCs, recently became the first targeted therapeutic cancer vaccine to be approved by the US Food and Drug Administration (FDA). However, ex vivo therapies such as Provenge have practical limitations and elicit an immune response with limited scope. By contrast, live-attenuated Lm naturally targets DCs *in vivo* and stimulates both innate and adaptive cellular immunity. Two different Lm-based vaccine platforms have advanced into phase II clinical trials in cervical and pancreatic cancer^35^. The possibility of employing engineered Lm to establish HCV pan-genotypic protection is promising based on the studies which have employed Lm-based DC-targeting vaccines to induce simultaneous responses to multiple antigens^35^. Future Lm-based clinical vaccine candidates are expected to feature polyvalent antigen expression and to be used in combination with other immunotherapies to augment efficacy.

Another major challenge for a therapeutic vaccine is the poor innate and adaptive cellular responses observed in the setting of chronic infection, such that even what should be an effective vaccine might limited in virally infected individuals simply because of diminished cellular responses, likely due to virus-mediated negative signaling molecules [27]. Specifically, chronic HCV infection is associated with exhausted CTL responses [5]–[8], and a defective antiviral CTL response is critical to HCV persistence, treatment response, and vaccine development. In addition, induction of an effective immune response requires the active participation of host APCs, the most potent of which are DCs [2]–[4]. Strategies enhancing DC function to improve CTL response have been reported to be effective in overcoming immune tolerance in antiviral and antitumor therapeutic vaccines [25]–[27]. Recent studies indicate that Tim-3 plays a pivotal role in impairing innate to adaptive immune responses during chronic HCV infection [40]–[41], [44]. This inhibitory receptor could indeed interact with its natural ligand, Galectin-9, expressed by monocytes, Tregs, and HCV-infected hepatocytes, or with phosphatidylserine expressed by dying cells. Moreover, apoptosis of exhausted CTLs may be induced by LM-NS5B/DCs [27], [40]–[44]. Additionally, exhausted CTLs co-express various exhaustion receptors (including PD-1, CTLA-4, or CD160) during chronic infection [40]–[44], and thus it is worthwhile to explore whether blockade of other inhibitory pathways or dual blockade may trigger the same or even more extensive expansion of HCV-specific T cells. In this study using an Lm-HCV expressing DC-targeting model system, we show that Tim-3 is highly expressed on monocyte-derived DC as well as T cells, and blockade of Tim-3 signaling can significantly improve the capacity of DC uptake antigens, secretion of Th1 cytokines, and triggering of virus-specific CTL responses. Notably, merely inhibition of Tim-3 signaling during DC maturation or in T cells was not sufficient to drive the expansion of HCV-specific T cells, suggesting that Tim-3 pathway suppresses innate as well as adaptive immune cells, and a good CTL response requires rescue of both APCs and T cells, including virus-specific CD4^+^ as well as CD8^+^ T cells. Indeed, Tim-3 was found to be expressed high on CD4^+^ T cells, concomitant with a lower level of IL-2 expression by these cells, from HCV-infected versus uninfected subjects (data not shown). Since Tim-3 blockade was carried out with DC induction as well as incubation with total CD3^+^ T cells, the role of helper CD4^+^ T cells must be considered in count of the improved CTL responses. This study indicates that novel strategies to enhance the potential of both innate and adaptive immunity by improving antigen presentation and cellular responses are essential for generating an effective therapeutic vaccine against chronic infection.
